# Capillary Leak Syndrome Aggravated by Influenza Type A Infection

**DOI:** 10.7759/cureus.2554

**Published:** 2018-04-30

**Authors:** James L Lawrence, Hussam Hindi

**Affiliations:** 1 West Virginia School of Osteopathic Medicine, Frederick Memorial Hospital, Lewisburg, USA

**Keywords:** capillary leak syndrome, influenza, hypotension

## Abstract

A 41-year-old female presented to the emergency department with nausea, vomiting, and diarrhea. Five days prior to this, she tested positive for influenza type A in an urgent care clinic and received Tamiflu. She also complained of generalized weakness in her extremities. Her initial labs were concerning for a grossly elevated hemoglobin and hematocrit despite adequate fluid resuscitation. Her condition continued to worsen as she developed distal cyanosis in all of her extremities and pulselessness. She was treated with IV hydration, bicarbonate, heparin, vasopressors, albumin replacement, Tamiflu, and phlebotomy. Her blood pressure continued to deteriorate rapidly. The arterial blood gas (ABG) depicted a case of severe metabolic acidosis that ultimately resulted in respiratory failure, and she required mechanical ventilation within 24 hours. Systemic capillary leak syndrome is a rare disease that is characterized by hypotension, hemoconcentration, and hypoalbuminemia. In this instance, influenza type A played a large role in its development.

## Introduction

Dr. Bernard Clarkson described capillary leak syndrome in 1960 in a patient with sporadic episodes of hypotension, edema, and hypovolemia [1]. Since then, there have been approximately 412 total cases of systemic capillary leak syndrome published in the literature [2]. The rarity of systemic capillary leak syndrome has limited the amount of research and knowledge about its pathogenesis and treatment. The pathogenesis is believed to be related to dysfunction in barrier endothelial cells leading to the leakage of fluid from the intravascular space [2]. At this time, the most common treatment modalities are intravenous (IV) fluids and vasopressors to reduce hypoperfusion to vital organs and tissues. Here, we describe the case of a rapid onset of polycythemia, hypotension, and hypoalbuminemia in a patient who was diagnosed with influenza type A treated with Tamiflu five days earlier.

## Case presentation

A 41-year-old woman with no significant past medical history presented to the emergency department with a one-day history of nausea, vomiting, and diarrhea (six loose bowel movements within the previous 24 hours). Five days prior to her presentation, she presented to an urgent care clinic with body aches, chills, and fever. She tested positive for influenza type A and was prescribed Tamiflu. Her husband and several other family members also tested positive for influenza. On admission to the emergency department, she denied chills, fever, abdominal pain, numbness, or paresthesias. Nausea and vomiting were exacerbated by oral intake of food and fluid. She also complained of generalized weakness in her upper and lower extremities bilaterally, as well as shooting pain down the lateral side of her right lower extremity. On physical exam, she was noted to have dry mucous membranes. The rest of her exam was unremarkable. Vital signs on admission were a temperature of 95.5° F, pulse 94/min, respiratory rate 16/min, and blood pressure of 121/58 mmHg. She was started on 0.9% intravenous sodium chloride and Tamiflu. She was also started on ondansetron, prochlorperazine, and diphenhydramine for intractable nausea and vomiting. Her initial labs showed the following: white blood cell count (WBC) 13,200/mm^3^, hemoglobin (Hb) 21.5 g/dL, hematocrit 67.3%, platelet count 361,000/mm^3^, sodium 135 mEq/L, potassium 5.8 mEq/L, chloride 96 mEq/L, blood urea nitrogen 16 mg/dL, creatinine 1.1 mg/dL, glucose 169 mg/dL, calcium 8.4 mg/dL, and lactic acid 7.5 mg/dL.

After IV fluid administration, she stated she was starting to feel better, but this was short-lived. She continued to have generalized muscle pain, weakness, and feelings of heaviness in her lower extremities. At this time, there was no evidence of edema. Repeat labs showed continued elevated hemoglobin and hematocrit levels; therefore, phlebotomy was recommended. Hours later, the patient became lethargic and experienced numbness and tingling in her hands and feet. On generalized exam, her extremities appeared purple and cyanotic. She denied pain but once again described pressure in her lower extremities. Her pulses were unable to be obtained by Doppler ultrasound. She was transferred to the Intensive Care Unit where an arterial blood gas (ABG) depicted a pH of 7.148, pO2 146 mmHg, pCO2 25 mmHg, and a base excess of -18. Her albumin level was 2.4 g/dL, and she was started on replacement therapy. Her creatine phosphokinase (CPK) levels and liver function test were within normal limits. Neurology was consulted after episodes of sensory deficits to rule out Guillain-Barre syndrome.

On physical exam, she continued to have generalized weakness but presented with hyperreflexia of her deep tendon reflexes. It was recommended that a lumbar puncture be used to evaluate albuminocytologic dissociation and to possibly administer intravenous immunoglobulin (IVIG). After discussion, it was agreed that Guillain-Barre syndrome was unlikely and that further workup was not necessary. Labs showed no evidence of methemoglobinemia or carboxyhemoglobinemia. Heparin, insulin sliding scale, prochlorperazine, sodium bicarbonate, and IV fluids were administered. Her blood pressure began deteriorating rapidly, so Neo-Synephrine® was administered and the required doses of Neo-Synephrine escalated quickly. Repeat labs showed a continued elevation of the hemoglobin and hematocrit. Her WBC count increased significantly to 42,000/mm^3^. Labs confirmed that metabolic acidosis due to lactic acid continued to be present. She required intubation due to respiratory failure induced by her metabolic acidosis. She was coherent before intubation, and she only complained of generalized weakness and pressure on her lower extremities. Peripheral access was needed to run more lab work but was impossible due to the collapsibility of her veins. A central line was planned. However, the patient went into asystole and cardiopulmonary resuscitation (CPR) was attempted to resuscitate the patient to no avail.

## Discussion

Systemic capillary leak syndrome is characterized by massive fluid and protein extravasation from the intravascular space into the extravascular space with symptoms of hypoalbuminemia, hemoconcentration, and hypotension without secondary causes. The hypothesized pathogenesis of capillary leak syndrome is an activation of T-cells in response to certain triggers, such as viral infections, that leads to diffuse cytokine activation which causes impairment in endothelial cell barrier function throughout the body [2]. Elevated levels of vascular endothelial growth factor (VEG-F) and angiopoietin-2 (Ang2) are also thought to contribute to the pathogenesis of capillary leak syndrome by increasing vascular permeability and damaging endothelial cell barrier stabilization [3]. Capillary leak syndrome’s initial prodrome of myalgia, occasional fevers, vomiting, diarrhea, and abdominal pain is followed by the rapid onset of hypotension, edema, and the third-spacing fluids. This is termed as the leak phase [4]. The massive edema and hypotension typically lasts for about two to three days and then is followed by a post-leak phase consisting of fluid reversal. In the post-leak phase, massive amounts of fluid held in tissues move into circulation. This carries a risk of fluid overload secondary to possible overzealous fluid resuscitation that can result in mortality [4].

There are many distinguishing features of capillary leak syndrome that should separate it from other possible etiologies of hypotension and edema. Polycythemia vera has similarities in its features of elevated leukocytes, hematocrit, and hemoglobin levels. However, unlike capillary leak syndrome, polycythemia vera is often characterized by normotensive patients without edema. Dehydration is another differential diagnosis due to its common findings of hypotension and volume depletion with capillary leak syndrome. However, dehydration responds appropriately to IV fluids. In capillary leak syndrome, IV fluids are not able to quickly reverse hypotension, and the hematocrit concentration often rises after IV fluid administration due to continuous leakage of intravascular fluid into extravascular space. Septic shock can present in a similar pattern to capillary leak syndrome, although septic shock commonly has a normal albumin with a suspected source of infection. Anaphylaxis has overlapping symptoms with capillary leak syndrome, such as acute hypotension and edema. However, anaphylaxis commonly has a normal albumin, urticarial rash, and an elevated tryptase [4].

Immediate treatment of acute capillary leak syndrome focuses on symptomatic treatment of the patient’s hypotension, hypoalbuminemia, and hemoconcentration with IV fluids, vasopressors, albumin, and bicarbonate. Preferred IV fluid treatment is that of rapid colloid boluses of 25% albumin rather than continuous crystalloid infusion to maintain hemodynamic stability in the patient [4]. Fluid resuscitation should not be aggressive at achieving normotensive pressures since the underlying pathology results in third-spacing of fluids and risks the development of fluid overload and pulmonary edema [4]. Rather, resuscitative treatment should acknowledge that hypotension, oliguria, and lactic acidosis are expected, and the treatment should be catered towards preventing rapid hemodynamic deterioration [4]. Intravenous immunoglobulins have been used in prophylactic treatment and in the acute treatment of systemic capillary leak syndrome. Lambert et al. documented the efficacy of IVIG in reducing symptoms in three cases of acute systemic capillary leak syndrome, although not all cases of documented capillary leak syndrome have shown an improvement from IVIG [5]. Other possible treatments of capillary leak syndrome include theophylline and terbutaline. The hypothesized reasoning for using theophylline and terbutaline as treatments is that they stabilize vascular endothelial (VE)-cadherin-mediated adhesive junctions of endothelial barriers [3].

## Conclusions

Capillary leak syndrome secondary to influenza A virus in an adult has only been well-documented once before in a 2016 case report. This case report describes a probable diagnosis of capillary leak syndrome secondary to influenza A virus with clinical manifestations of metabolic acidosis, diffuse hypoperfusion, and respiratory failure. The patient's influenza A viral infection likely triggered the sudden onset of capillary leak syndrome. The patient’s prodrome of myalgia, vomiting, diarrhea, and nausea, followed by the rapid development of hypotension, hypoalbuminemia, hemoconcentration, and third-spacing of fluids, is congruent with the leak phase of capillary leak syndrome discussed above. This case report raises awareness for the potential complication of capillary leak syndrome aggravated by influenza A viral infection. Future physicians can look for the rapid onset of hemodynamic deterioration with hypotension, hypoalbuminemia, and hemoconcentration as clinical signs that capillary leak syndrome may be a potential pathology. The underlying influenza A viral infection also may have impacted the clinical progression of capillary leak syndrome due to the heightened cytokine inflammation from the infection itself. With a preceding influenza type A infection, this patient’s risk of elevated anion gap metabolic acidosis was even higher due to repeated episodes of diarrhea and vomiting leading to a state of volume depletion from the infection. This case report explores the clinical presentation of capillary leak syndrome and the potential treatment options for such a rare disease.
